# An evolutionary perspective on gradual formation of superego in the primal horde

**DOI:** 10.3389/fpsyg.2014.00008

**Published:** 2014-01-24

**Authors:** Erdem Pulcu

**Affiliations:** Neuroscience and Psychiatry Unit, University of Manchester Medical SchoolManchester, UK

**Keywords:** primal horde, superego formation, sublimation, fire, evolutionary psychology, morality, behavioral economics

## Abstract

Freud proposed that the processes which occurred in the primal horde are essential for understanding superego formation and therefore, the successful dissolution of the Oedipus complex. However, Freud theorized superego formation in the primal horde as if it is an instant, all-or-none achievement. The present paper proposes an alternative model aiming to explain gradual development of superego in the primitive man. The proposed model is built on knowledge from evolutionary and neural sciences as well as anthropology, and it particularly focuses on the evolutionary significance of the acquisition of fire by hominids in the Pleistocene period in the light of up-to-date archaeological findings. Acquisition of fire is discussed as a form of sublimation which might have helped Prehistoric man to maximize the utility of limited evolutionary biological resources, potentially contributing to the rate and extent of bodily evolution. The limitations of both Freud's original conceptualization and the present model are discussed accordingly in an interdisciplinary framework.

Et tu, Brute?!

Julius Caesar, William Shakespeare

## Introduction

Freud was one of the first theoreticians to elaborate on the evolutionary mechanisms in human psychology. This viewpoint was partially driven by the influence of the Lamarckian theory on Freud's conceptual work (Gershenowitz, 1978). Freud supported the Lamarckian idea of “inheritance of acquired characteristics” as means of linking his observations obtained at the patient level to those innate biological characteristics idiosyncratic to humans, which are acquired during the course of evolution. In his later writings, Freud adopted Haeckel's recapitulation theory (Gould, 1977) explained by the famous notion of “ontogeny recapitulates phylogeny.” Recapitulation theory extended beyond early 20th century and the borders of the embryonic development, on which it was initially based. For example in neurology, MacLean proposed that the human brain consists of layers which date back to different evolutionary origins (Holden, 1979; MacLean, 1985). Recent advancements in cognitive neuroscience support this hypothesis, associating the limbic system with a more primitive evolutionary origin (Mega et al., 1997), whereas the prefrontal cortex; which is a hub of rational decision making and top-down control over the limbic system (Knoch et al., 2008) is proposed to have evolved much later, both phylogenetically and ontogenetically (Giedd, 2004). Haeckel's theory of recapitulation influenced Freud to move toward a Darwinian approach from his initial Lamarckian viewpoint, leading him to explore the adaptive functions of psychological mechanisms (Nesse and Lloyd, 1992) and their significant disturbance in psychopathology (Freud, 1923, 1926).

Among these psychological mechanisms the evolutionary function of conscience and/or superego is particularly interesting. Freud investigated the development and the functions of superego at two levels; development of superego in the child and the ability of superego formation as acquired by mankind during the course of evolution. According to psychoanalytical theory, development of superego in the child is a gradual process which is resolved by internalization of the father's norm (as well as normative behaviors) and subsequent repression of sexual urges cathected toward the mother leading to a successful dissolution of the Oedipus complex (Freud, 1924).

Overlaid onto the conscious and unconscious realms in the topographical model (Freud, 1900), superego develops gradually and acts as an agent regulating the expressions of instinctual needs by the reality principle. Freud developed the regulatory role of superego in the structural model in three successive essays between 1920 and 1926; in *Beyond Pleasure Principle* (Freud, 1920), *Ego and Id* (1923), and *Inhibitions, Symptoms and Anxiety* (1926). As a part of this regulatory role, superego censors *Trieb*-driven information (which originates from the id) from the ego. In its affective repository, superego primarily makes use of fear of punishment and derivative anxiety on one hand and anticipated guilt on the other (Payne, 1927), when demands of the id find expression behaviorally; bypassing regulations with the familial and societal norms. Moral beliefs and particularly moral emotions (e.g., guilt, shame, gratitude, indignation etc.) are important in terms of regulating behavior and balancing selfish needs with those accepted by societal norms, both in health (Tangney, 2002; Tangney et al., 2007) and in psychiatric conditions; in which there is abnormality in processing of these emotions (O'Connor et al., 2007). Therefore, modern understanding of superego as an observing agency may posit that it regulates behavior in accordance to dynamic societal norms and internalized borders of personally acceptable behavior drawn by subjective degrees of anticipated guilt and fear of punishment. Global evaluation of behavioral research indeed suggests that guilt has an important role in motivating morally congruent behavior, both in the acute affective phase as well as when it is anticipated (see review of this literature in Pulcu et al., 2013).

The focus of this paper is the different set of criteria Freud employed for the development of superego in the primitive man of the primal horde, in an attempt to shed light on how humans acquired this capacity of self-regulation. Freud discussed the superego formation in the primal horde as an achievement which is a product of an instant dissolution. This controversy constitutes the particular subject of this manuscript. The first section of this paper will explore the formation of psychological self-regulatory agency (i.e., superego) in the primal horde from an evolutionary perspective. The second section examines the evolutionary significance and the psychoanalytical meaning of [the acquisition of] fire (perhaps, one of the most important of all human achievements). The potential link between the acquisition of fire and superego formation will be discussed accordingly, aiming to provide an evolutionary model in which superego may have emerged gradually in the primal horde. Due to reconstructive nature of the present discussion, although mostly unavoidable, it may be regarded as speculative by some of its audience. This is explicitly addressed in the limitations section. In order to address limitations associated with a theoretical-reconstructive, rather than a data-based approach; some of the technical and ethical limitations restricting formulation of empirically testable hypotheses will also be discussed.

## The primal horde

Freud's views on the primal horde are among the first evolutionary writings on human psychology. The concept of primal horde is constructed upon a similar concept which is called the “primitive horde” used by Charles Darwin, referring to the simplest possible form of a social group of mammals. On the other hand, the primal horde refers to a primitive group of Homo sapiens in which a powerful male ruled despotically over the rest. Due to his physical fitness only the male leader had access to sexual gratification. The descendents (sons) were forced to submit to the will of the leader (primal father) or to confront him. At some point along the course of time the primal father was killed by the sons to take his possession. According to Freud, the internalized traces of remorse and guilt following this act became the essence of conscience and superego. Freud (1913) says:

“[the primitive men] hated their father, who presented such a formidable obstacle to their craving for power and sexual desires, but they loved and admired him too. After they got rid of him, had satisfied their hatred and had put into effect their wish to identify themselves with him, the affection which had all this time been pushed under was bound to make itself felt. It did so in the form of remorse. A sense of guilt made its appearance, which in this incident coincided with the remorse felt by the whole group. The dead father became stronger than the living had been.” (p. 143)

The condition of the sons in the primal horde, struggling between sexual frustration and fear of the primal father, seems to resemble the positioning of ego between id and superego in the structural model (Freud, 1923). Therefore, it is possible that Freud might have formulated hypotheses about the primal horde in order to give a holistic account of superego formation within psychoanalytical meta-theory.

In the same essay Freud (1913) highlights the oral cannibalistic mechanisms accompanying the identification. It is claimed that the sons have devoured the flesh of the primal father to internalize a part of him, leading to a complete psychological identification. Freud's view on the terminal resolution of the primal horde scenario is implicitly challenged for example by Lantos (1958) who provided a meticulous discussion of aggression and rejected any expression of oral aggression as an ultimate resolution of rivalry. It is claimed that neither self-preservative nor sexual instincts would guide an individual to express oral aggression after killing, as killing terminates rival aggression. Freud also suggested that, the totem animal replaced the primal father in time as a materialized symbol, which was then killed ceremonially as a commemoration. Thus, Freud puts the murder of the primal father at the bottom of all civil achievements, moral development and religion.

Although, Freud (1921) clearly stated the hypothetical nature of his assumptions regarding the primal horde, its relevance with the Oedipus complex and the superego formation is presented as if it is an all-or-none achievement. Especially, the guilt emerging after the crime and the collaborative nature of the act calls for attention. The group behavior and affective processing of behavioral consequences suggest that the conditions of the primal horde in Freud's hypothesis were already quite distinct from similar cases of animal hordes. In this respect, one should consider the presence of psychological mechanisms in the primal horde situation in contrast to purely biological mechanisms in any resembling animal hordes. Thus, Freud's primal horde should be placed further up on the evolutionary timeline relative to Darwin's primitive horde.

Both in Freud and Darwin's reconstructions, tension driven by sexual frustrations in the closed social group leads to physical challenges between the dominant male and the remaining males who are competing for group leadership. The father, with his immense influence on the female members of the horde, had an exclusive access to sexuality. From the female point of view, submission to the dominant male who exhibits cues of physical, reproductive and survival fitness is evolutionarily rewarding. Getting pregnant from the dominant male is the evolutionarily stable strategy^1^ for any female in the horde (see footnote). From the male point of view, challenging the dominant male is the evolutionary stable strategy for the rest of the males who have to confront the dominant male in order to gain access to sexual reproduction. The dominant male defends his acquired position by forcing the rest of the males to take a flight response, and flee from the horde. This view is expressed by Freud (1913) as a strategy to prevent close-interbreeding in the group. Dawkins (1976) who holds a similar opinion claims;

“despite the uncertainties that await those who leave, it is a less of a gamble compared to staying, for those who stay have to stick with fight strategies to challenge to dominant male at the cost of their lives.” (p. 119)

Consequently, the individual evolutionary economics of staying favors saving any possible investment (through parental investment at the beginning of life and avoidance of reckless encounters after the clinical developmental period) to grow bigger and stronger to challenge the dominant male at some point. Otherwise the genes of the males which avoid physical confrontations with the dominant male would not be represented in the future genetic pool.

Optimizing fight or flight responses is a matter of concern for only those individuals/cubs that survive through the critical developmental period. During the critical developmental period, successful attachment between the offspring and the mother is the utmost important factor in determining the survival fitness of the offspring, in which the offspring mostly has a passive role. Attachment theory (Bowlby, 1977; Bretherton, 1985) is biological in the sense that it studies mother-infant attachment in the critical developmental period and the impact of the way certain skills are mastered during this period on later life [see (Holmes, 2011) for theoretical and clinical perspective about how different mother-infant attachment styles influence the nature of superego in later life]. It is important to point out that although developmental course of humans and nonhuman animals follow a similar course during the critical period, these are not directly comparable as the human bear children prematurely relative to nonhuman animals; which is considered as an evolutionary cost of achieving bipedal locomotion (Whitcome et al., 2007). Therefore, once the mother-infant affective bond is formed, the females in the primal horde, as well as in nonhuman animals, must be hardwired to provide a secure attachment with their offspring so that offspring can master important survival skills which would directly influence the extent of how much of parents' genes will be available in the genetic pool in subsequent generations. Considering this point from a genetic perspective (Dawkins, 1976), evolution would not favor diversity in attachment styles in nonhuman animals [which may produce hyper-or-hypo vigilant individuals (Holmes, 2011) and these may have poor survival likelihood relative to those individuals who follow a normal developmental course], it is possible to speculate that the primal horde scenario might have occurred before humans mastered bipedal locomotion, when human behavior was more biologically determined. This view suggests that although different attachment styles contribute to how people perceive their social/physical environments and master coping skills adaptively (Holmes, 2011), these did not compromise immediate survival fitness of growing offspring in the critical developmental period given that human evolution favored diversity in this domain.

In the next step, it is important to address the problem of interbreeding faced by those individuals who followed a normal developmental course and out powered the primal father in a physical competition. The problem of interbreeding has an effect on the overall fitness of the horde in relation to horde's size. A negative correlation is predicted between the size of the horde and possible detrimental consequences of interbreeding. When the size of the horde is reduced to a single family a classical phenomenon of Oedipus complex is observed. In psychoanalytical theory, the father as the representative of the societal law prohibits the son's sexual desires targeted upon the mother. Thus, the predominant tendency to take a flight from the family and to find spouses through competition with other individuals who do not have close a kin relationship seems to be an achievement of mankind. This behavioral strategy contributes to the terminal resolution of the Oedipus complex.

The following case is an example of a primal horde demonstrating the possible consequences of close interbreeding. After the death of the primal father, incest might have been a common practice in gratification of sexual desires. As a result, the detrimental effects of close interbreeding were observed in the offspring. An inverse relationship is predicted between the frequency of incest and the life expectancy of the offspring as the conditions of prehistoric ages demanded physical fitness for survival. The exquisite knowledge of the consequences of close interbreeding and the works of recessive alleles were not available to the prehistoric man. It is possible that he was superstitious in making causal attributions for environmental uncertainties. The deficiencies observed in the offspring might have been attributed to the curse of the primal father who was killed to gain access to sexual gratification. Therefore, the essence of superego should have grown gradually. At the end as Freud (1913) suggests, the primal father becomes more powerful after he is been killed. In Freud's model, the figure of the primal father and his curse become gradually omnipotent, replaced by the figure of God. In this reconstruction, the memes^2^ are observed as emerging out of genetic influences and it might be suggested that the memes for “thou shall not kill” and “thou shall not commit incest” probably were the first complementary replicators (see footnote). The present view also contributes to the understanding of the resolution of Oedipus complex.

In summary, Freud proposed two hypotheses with psychological nature related to the primal horde. The first hypothesis concerned the resulting guilt which emerged in relation to the murder of the primal father. The second one concerned the collaborative nature of the murder. In the animal hordes which have similar conditions to the primal horde (e.g., a horde of elephant seals in which a dominant male controls a harem of females) the successor replaces the dominant male. However, Freud presents his hypothesis as if there is a revolution against the law of the primal father: sons dethroning him. Freud's emphasis on group behavior separates the primal horde from the animal hordes. I have used a quote from Shakespeare's classical tragedy, Julius Caesar, at the beginning of this section. I think it beautifully illustrates formation of interpersonal networks to challenge an autocratic leader. In his final words Caesar utters his astonishment to see Brutus among the conspiring network. Although, Freud did not make any explicit connections with Julius Caesar, it may be a topic of interest for historians of psychoanalytical theory to explore whether Freud had considered the resemblance. The guilt produced as a result of killing the primal father also suggests that the primal horde went far enough on the evolutionary timeline to be distinct from the rest of the animal hordes. This is because actions motivated by life preservative instincts and drive for reproduction do not produce guilt in animals.

Another key defense mechanism which Freud treats systematically differently between the primal horde scenario and in his discussion of the dissolution of the Oedipus complex is repression. In psychoanalytical developmental psychology, repression of sexual attachment of the infant to the mother contributes to the successful dissolution of the Oedipus complex (Freud, 1924). On the other hand, Freud proposed that sexually motivated killing of the primal father produced a profound guilt in the primal horde, which is then commemorated annually by the killing of the totem animal. This view suggests that the primal horde functioned with a collective conscious that works against repression, particularly of the memory of the murder and possibly the associated sexual motivations. Freud's conceptualization of repression in the primal horde scenario also suggests that repression is an important aspect of societal development which contributes to cultural transformation that leads to formation of modern societies.

In order to fully grasp psychoanalytical processes in the primal horde, it may be important to put these processes in a temporal perspective. Challenge to the primal father suggests that behaviors at this stage are biologically driven as it may be in any other animal hordes. Therefore, the execution of behavior (i.e., successfully challenging the primal father) at this stage becomes a question of adequacy rather than failed inhibition. Freud did not make any explicit suggestions about the next component in this sequence, namely what happened sexually once the primal father is killed. Therefore, it is not possible to provide a reconstruction of this stage, but I have discussed the potential consequences of interbreeding above. According to Freud, physical challenge is followed by an affective response (i.e., guilt) which precedes inhibitory psychological mechanisms at this stage, such as repression. However, it is not possible dissociate affective and inhibitory processes from each other conclusively; aiming to address whether guilt is reactive to killing of the primal father or emerges retrospectively after observing biological consequences of interbreeding which are associated with the killing of the primal father. Consequently it remains a puzzling question, whether repression and other inhibitory mechanisms emerged *post-hoc* such that sequences of actions with detrimental effects are avoided unconsciously or *propter-hoc* in order to reduce psychological costs associated with anticipated guilt which inhibits morally incongruent actions. There is emerging evidence to suggest that guilt may be particularly important as evolutionary studies showed that agents with prosocial emotions were favored by evolution (Bazzan et al., 2002), emphasizing the importance of prosocial emotions in maximizing utility by devaluing short-term rewarding outcomes of self-serving decisions by providing additional psychological costs.

Repression is a psychological defence mechanism which is difficult to study empirically by neuropsychological testing batteries (see below for further discussion). However, it may have an important role in minimizing psychological costs, through denying awareness to motivations which may lead to aversive behavioral outcomes (Boag, 2007). Certain psychiatric conditions are associated with abnormal evaluation of anticipated behavioral outcomes in which anticipated affective responses devalue projected utility. Major depressive disorder is particularly interesting in terms of understanding the magnitude of such projected psychological costs through abnormal processing of guilt (American Psychiatric Association, 2000), in which the moral self-evaluation system is proposed to be on “overdrive” (O'Connor et al., 2007). In *Mourning and Melancholia*, Freud (1917) provided the first clinical account distinguishing depression from healthy forms of sadness by highlighting the presence of abnormal self-criticism “tormenting” the ego. Although Freud did not focus on the possible involvement of inhibitory mechanisms, hyper scrutiny of behavioral motivations and abnormal self-criticism in major depression may be related to the failure of repression, such that these unwanted thoughts become a cardinal object of depressive rumination (Brinker and Dozois, 2009).

Discussions about whether psychoanalytical processes observed in the primal horde and related ego defenses such as repression would produce testable hypotheses within moral/cognitive psychology framework is one of the key challenges in neuropsychoanalysis (Boag, 2007). Modern evidence-based neuroscience would support that Freud's primal horde should be distinct from Darwin's primitive horde as higher order cognitive processes such as guilt and repression, as well as a general moral organization of the human brain which relate to fronto-meso-limbic networks (Moll et al., 2005). These networks are regarded to have evolved later than the medial temporal regions, such as the amygdala, responsible for processing primary emotions and fight-or-flight responses. Although, neurobiological substrates of intra-family affective bonding, common to most of the mammals, are localized in the limbic regions which are regarded as phylogenetically more primitive (Young and Wang, 2004), key regions for healthy functioning of moral emotions, such as the anterior cingulate and fronto-insular cortices in fact contain neurons unique to humans (Seely et al., 2012). In the past two decades, neuroscientists have identified effective paradigms to decipher the function of the neocortex in moral/affective processing domains, such as the Trolley Dilemma (which make use of activating evolutionarily hardwired mechanisms for valuation of human life, Greene et al., 2001) and/or Emotional Stroop tasks (designed for understanding conflicting mechanisms during cognitive/affective processing, Banich et al., 2000), which could be used to study aggressive/sexual themes in hypothetical conflicts. Nevertheless, integrating intra-family member oriented scenarios in sexual/aggression domains to these paradigms may pose serious ethical considerations, consequently limiting their applicability as empirical investigation tools.

Although an exhaustive review of the consciousness literature is beyond the merit of this paper, it is important to mention some of the recent meta-analytical evidence about consciousness as repression disrupts the flow of information from the unconscious realm to consciousness. Recent evidence suggests that information processed consciously requires mental imagery of the anticipated situation, complex sequential behavioral planning and evaluation of potential consequences which relate to activations in temporoparietal junction, frontopolar and medial orbitofrontal cortices (Boly et al., 2013). These action sequences are unique to humans, such that in animals sequential behavior is mostly conditioned to obtain primary rewards, and in novel environments it is mastered through learning in repeated trial and errors (but not mental imagery), where mastery enhances connectivity between brain regions associated with rewards and behavioral planning (Schultz, 2004). Although there is an overlap in neural organization of consciousness between humans and nonhuman animals (Boly et al., 2013), utility-based evaluation of behavior through mental imagery of anticipated consequences is unique to humans. In this respect, consciousness not only involves biological spatial orientation increasing cost efficiency of motivated behavior and peripheral sensory feedback but also psychological awareness. Cytoarchitectural work started at the beginning of the century with Korbinian Brodmann's important contributions (1909), suggests that the outermost layers of the cerebral cortex implicated in mental imagery and sequential behavioral planning, such as the frontopolar and temporoparietal cortical regions, are indeed related to a later phylogenetic formation, distinguishing humans from other nonhuman animal species.

Due to the evolutionary approach of the present manuscript, it is important to point out to a few suggestions about the evolutionary functions of repression (Nesse, 1990). One leading suggestion also shared by some of the evolutionary biologists is that repression conceals motives in the sexual/aggression domains from oneself, and consequently concealing them even better from other individuals (Trivers, 1985; Alexander, 1987). Arguably, if the child is able to conceal such selfish motives successfully and delay short-term gratification, s/he would be able to benefit from parental investment for a longer period of time (Nesse, 1990). This perspective is compatible with purely biological motivations observed in resembling animal hordes as previously mentioned above (e.g., elephant seals). In fact, there is data to support that in humans, likelihood of parents getting murdered by their offspring increase as they age (Daly and Wilson, 1988) suggesting that parents are being devalued exponentially as they lack their resource provider roles, but the contribution of intra-family sexual motivations to such homicides would be highly debatable in the modern society. Both the psychoanalytical theory and the evolutionary biology conceptualize child's role in the family triad similarly; stating that the child is motivated to separate the parents. However, evolutionary biology posits that the motivation is not of sexual nature as put forward by the psychoanalytical theory, but it is about maximizing parental investment by deferring mother's labor as much as possible. In this respect, evolutionary biology provides a viewpoint which is applicable to all species of mammals that mostly produce heterozygotic cubs which rely on their mother's investment mostly until they reach sexual maturity and be able to survive on their own.

The next subsection explores the significance of fire in human natural history and discusses whether the acquisition of fire could be a form of sublimation driven by frustration of sexual impulses. Secondly, the conditional role of this sublimation in explaining how a transition from purely biological mechanisms to psychological mechanisms could become possible in the primitive man will be discussed. Taking this perspective, I aim to provide one possible reconstruction of the primal horde which allows gradual formation of the superego.

## Fire and its meaning in psychoanalytical literature

The acquisition of fire is probably one of the most significant achievements in human evolution (Burton, 2011). Therefore, prior to discussion of its psychoanalytical meaning it is important to provide a brief overview of anthropological and archaeological evidence regarding domestication of fire, suitable for the purpose of this manuscript. Studies conducted on fossilized charcoal samples suggest that wildfires started to occur as early as 420 million years ago (Ma) after terrestrial plants appeared in the Silurian period (Scott and Glasspool, 2006). The spread of highly flammable savannas changed the flora in Africa and this may have contributed to hominids' acquisition of fire (Ségalen et al., 2007). In the Mediterranean region, lightning has been suggested as the major cause of natural wildfires (Whelan, 1995). It has been suggested that initially such naturally occurring processes were used opportunistically to obtain fire for heating purposes and as an orientation point while travelling inland (Bellomo, 1994). Fossil studies claim that the evidence of cooking may date back to 1.9 Ma (Wrangham et al., 1999). However, reliable evidence of control over fire in the African hominids dates back to the Pleistocene period (Bellomo, 1994), although it has been suggested that early domestication of fire was restricted for use as a means of protection against predators and for lighting and heating (Bellomo, 1994). A more recent study claims that hominid evidence of control over fire for domestic purposes has been found in Gesher Benot Ya'aqov, Israel, dating back to 790,000 years ago (Goren-Inbar et al., 2004). In the light of these archeological findings, it may be proposed that the primal horde could date back to the early to middle Pleistocene period.

### Fire in psychoanalytical literature

Psychoanalytical interpretation of the significance of fire, could be discussed in three important domains: inhibition of an instinctual urge to extinguish fire when it is encountered, lighting fire by using stone objects which could produce spark and control of fire in a particular place and size. Psychological meaning of fire was first considered by Freud (1930a,b) in a footnote to *Civilization and Its Discontents*. Subsequently, Freud (1932) elaborated on his initial assumptions regarding the acquisition and control of fire:

“in order to gain control over fire, men had to renounce the homosexually tingled desire to put it out with a stream of urine.” (p. 187)

Freud's arguments were based on material derived from the myth of Prometheus who has stolen the fire protected by Gods and carried in a hollow stick. Erlenmeyer (1932) supported Freud's view by citing Genghis Khan's ruthless death penalties subjected to those who failed to renounce the instinctual passion for extinguishing fire. Regardless of the unconscious motive behind it, these authors who provided the first psychoanalytical discussions related to fire, agree that there is an instinctual involvement in extinguishing fire.

It is possible that there may be differences for what it is meant to be understood by “instinct” between Freud and contemporary psychoanalytical authors, particularly for discussions related to meaning of peripheral symbols, such as in this case; fire, which are not central to understanding fundamentals of the psychoanalytical theory. Freud in his seminal work, *Instincts and their Vicissitudes* (1915), made a distinction between *Instinkt* and *Trieb*, and used *Instinkt* predominantly, but not mutually exclusively, to define motivational aspects of animal behavior, whereas preferring *Trieb* mainly for driving forces of human actions. How well these differences in original German language is translated to the English audience remains a matter of debate, and there have been suggestions that the original work should have been translated as “Drives and Their Fate” (Mills, 2004), capturing the temporal dynamics of drives: initiating from the unconscious, guiding/motivating the behavioral toward an aim and extinguishing once the driving need is fulfilled as Freud intended to frame. Although, differences between translations are important to mention as a part of the present discussion, their terminal differentiation is not fundamental to understanding its core message. It may be useful to point out that Freud did not use *Instinkt* or *Trieb* in the *Civilization and its Discontents* footnote, but instead he initially preferred *Lust* when defining man's inner urge to extinguish fire in the German original (p. 47), which is translated as “desire” in the *Standard Edition*. On the other hand, in subsequent sentences he used *Trieb* in the same context for man, but preferred *Lust* again when defining how anatomical differences contribute to behavioral expression of such motivations differently in women (also see below). The slight variations within the same footnote suggest that concepts related to drives, motivations and instincts are not well differentiated, and are used almost interchangeably within the discussion related to psychological meaning of fire.

It is important to point out that some of the difficulties in translation of “*Trieb*” to English-language as “instinct” might have originated from Lamarckian influence on Freud's early manuscripts and Freud's well-known dedication to found psychoanalytical theory in human biological psychology which might have biased early translators of his work (who were also trainees of psychoanalysis themselves), to overemphasize the link to biological sciences. Lamarckian understanding of instinctual animal behavior involves stereotyped, species typical fixed action patterns which may be transmitted from one generation to another through inheritance of acquired characteristics, such that an offspring is biologically hardwired to act in a certain way, for example in order to get ration from the environment, even if it does not have any opportunity to learn such behavior from its mother. Due to the complexity of human behavior, even in instinctual domains such as sexuality; Freud did not use instinct (i.e., *instinkt*) to define human motivated behavior, but instead preferred *Trieb*. This distinction implicitly suggests that Freud conceptualized human motivated behavior not as stereotyped and hardwired in many aspects as may be in other species. I think the ongoing debate in the psychoanalytical field partially arise from initial shortsighted translations of Freud's work; presumably the early translators did not foresee that subtle nuances lost or gained in translation would remain to be a matter of debate 100 years later. Considering that *Trieb* in Freud's conceptualization contains unconscious somatic driving forces for motivated behavior (not necessarily restricted to instinctual domains such as sexuality and aggression), presence of which could only be traced back to its source by psychoanalysis of observable behavior in a systematic manner (1915), it is possible that English translations considered “instinct” to be the most accurate word to highlight somatic/biological component captured by the word *Trieb*.

Freud and Erlenmeyer's seminal assumptions cannot be validated within the evolutionary biological theory as fire stands as a common danger for all the members of the animal kingdom. Therefore, their proposed instinctual behavior cannot have hardwired biological foundations. In the same footnote to *Civilization and Its Discontents*, Freud also had differentiated sexes in relation to the yield of this instinct. It was suggested that males have to renounce the instinct to extinguish fire in order to tame it, where as females due to their anatomy were appointed as the guardians of fire. From a biological perspective the instincts are innate and indiscriminate for both sexes. In this respect it might be argued that females were excluded from acquiring such an instinct like the rest of the animal kingdom. From male's perspective, Freud's account implicitly suggests an ambivalent attitude toward fire: an urge to extinguish it in the presence of an opposing urge to keep it alive by appointing women as guardians. Therefore, the most plausible explanation/interpretation which provides theoretical consistency is that man's relationship with fire may be an object relationship in which fire symbolizes an external object, which is dominated by component instincts of sexual instinct in reference to an erotogenic zone. On this issue Freud (1932) says;

“The primitive man was bound to regard fire as something analogous to the passion of love.” (p. 190)

The instinctual associations of fire have been discussed further in psychoanalytical literature, but there is paucity of discussion in the field particularly about how the psychological meaning of fire may have changed over the course of human evolution. Stone (1979) suggests that oral aggressive impulses, such as the ones expressed by Freud in the resolution of the primal horde scenario, may be symbolically expressed by fire. There is some evidence to support pregenital involvement in male's relationship with fire in the psychoanalytical literature. For example, besides highlighting its oral significance, Grinstein (1952) presents fire as both a direct expression of aggressive and hostile impulses as well as libidinal ones, and conceptualizes the control over fire by retaining it in a particular place and time, as a symbol of the anal pleasure dominant in the second part of the anal stage. Therefore, there seems to be an agreement in the psychoanalytical literature about primacy for symbolic value associated with fire.

Some of the psychoanalytical authors went to extremes by claiming that fire could explain anything [symbolically] (Bachelard, 1964,p. 7). Such a global and overgeneral approach which is detected in one of the earliest accounts of “psychoanalytical” investigation of [the meaning of] fire, might have contributed negatively to general scientific credibility of the psychoanalytical theory (which in itself is so rich to formulate testable hypotheses), historically in an era where behavioral sciences were flourishing, due to lack of scientific rigor in handling the subject matter.

In order for an object to acquire a symbolic/psychological meaning, it has to be readily available in the environment. The object must be encountered either frequently or scarcely in a traumatic manner in order to produce long-lasting mental representations. Best to my knowledge, there is no anthropological or archaeological evidence to account for how frequently Prehistoric man encountered fire caused by natural events. Therefore, it is not possible to handle the contribution of repetition effects in a scientific manner any further than highlighting these principles. Acquisition of fire could be possible either in the stage of controlling dying wildfires in a particular place and size or lighting fire by using tools such as stones which could produce spark. Both of these stages require inhibition of an urge to put out fire, particularly in critical stages before skills to domesticate fire has not been acquired successfully. Otherwise, in order to gain experience with fire, Prehistoric man may have to wait until the next occasion in which a wildfire occurs within travelling distance, considering the evidence which suggests that experimentation with wildfires preceded tool based production of fire in the Stone Age, as reviewed in the beginning of this section. I think those authors who formulated that a symbolic value for fire may exist in the Prehistoric man are mostly anachronistic, such that it is not possible for fire to retain a symbolic value in humans without repeated safe, infrequent traumatic or repeated traumatic encounters with it (if it had continuously been extinguished instinctually) and it is not possible to formulate the urge to extinguish fire as a biological drive which exists mutually exclusively in human males.

On the other hand, it is highly likely that the primal father had these repetition properties, and could have acquired a symbolic value prior to fire. In the primal horde, the sexuality of the sons was denied gratification by the physically fit primal father. Therefore, repeated physical encounters must have existed between them, enough to create aversive mental representations in individuals who challenge the primal father physically. As widely accepted, sexual frustration creates aggressive impulses. Coincidentally and unintentionally one member of the primal horde might have manifested these two forces in operation symbolically and created fire:

“He, who created it, perhaps knocked two stones to manifest his aggression toward the father who frustrated him or created it by friction.” (Grinstein, 1952; p. 420)

This indeed symbolizes the sexual act or the primal scene (with one stone representing the primal father). This formulation by Grinstein is particularly interesting and pairing the acquisition of fire with the processes in the primal horde may be important for reconstructing one possible scenario which allows gradual formation of superego through repeated sublimations of sexual frustration. Meta-evidence suggests that using gestures in communication is mostly common across different species of higher order primates, but tool use in the wild is more restricted (van Schaik et al., 1999). However, if in reality the primal horde contained intermediate species between higher order primates and *Homo sapiens*, it would be highly likely that those species, although they may be extinct today, too had behaviors in their repertoire which contained some elements from both of these domains (i.e., gestures and tool use). Modern developmental psychoanalytical practice suggests that children's play contain variety of behaviors and meaningful preferences for specific toys which have symbolic value, providing material for psychoanalytical therapy (Klein, 1997). Gestures and use of mobile objects, such as the two stones suggested by Grinstein possibly representing a male and a female figure in the primal scene or physical challenges between the male members of the groups, become especially important when the subjects have limited repertoire for verbal expression. This may be a common denominator for both children in early course of their lives, higher order primates and the prehistoric man. Recent neuroscientific evidence provide support for this view, showing that people suffering from post-stroke aphasias, particularly when stroke affects speech production regions in the brain (i.e., Broca's aphasia), display elevated gestures relative to a healthy subjects in order to compensate for decreased capacity for verbal communication (Sekine and Rose, 2013). Similar tactile movements if combined by using stone objects which could spark in communication, might have contributed to lighting fire which is likely to be accidental in the first occasion. This constitutes the second important step toward acquisition of fire, by which Prehistoric man could learn to reignite fire even if it is blown out by external forces.

The final step in the sequence of events which lead to acquisition of fire is the ability of controlling fire in a particular place and size, in order to capture its most useful properties for everyday use. Transition from observing how fire lights up from sparks and investment of time to understand its properties, as well as symbolic representation of sexual or aggressive encounters in the primal horde (by knocking stones together) could be formulated as initial forms of sublimation, as the individual who accomplished this, must have diverted his attention away from immediate instinctual frustrations. This is how Freud (1915a,b), and Nietzsche (1878) earlier, conceptualized sublimation: as “engagement in activities which divert frustrated sexual energy into creative ends” (Gemes, 2009). Grinstein's reconstruction of the primal scene (see above) is a vivid example of how transition from frustrated sexual impulses could be invested in to understanding the nature of aversive elements, such as in this case fire. However, there are other psychoanalytical authors who suggested sublimation particularly concerns instinctual frustrations in the pregenital stages as genital sexuality is either repressed or satisfied due to the dominance of pleasure principle in this stage (Deri, 1939). Therefore, conceptually, it must be excluded from the scope of sublimation. As stated previously, Grinstein (1952) formulated that there may be pregenital involvement in the final stage of taming fire, particularly about attention dedicated to maintaining fire burning and keeping it at a safe size. This raises the question whether regression to earlier psychosexual development stages may lead to expression of sublimation in different ways, producing behaviors related to conservation, such as in the case with fire (or for example conservation of historical items/arts in the modern world).

Evaluating acquisition of fire as a form of sublimation with high evolutionary significance is compatible with both psychoanalytical theory and evolutionary biology. Once any given member of the primal horde acquired skills to produce fire repeatedly by using sparking stones and to control it safely in a particular size, it is plausible that it might have contributed to his survival fitness in physical confrontations with the primal father. If there is an imbalance in the level of skills related to the use of fire, such that the primal father lacked this knowledge in a physical challenge, it is likely that he would submit to the new authority or flee from the horde.

Application of behavioral economics is common to both evolutionary biology and psychoanalysis (particularly important in understanding secondary gains from neuroses observed in some patients; see Freud, 1923, p. 50). The acquisition of fire equipped prehistoric man against environmental dangers such as predators, night and cold weather:

“The acquisition of fire produced subsequent secondary gratifications of a kind which aided the development of further sublimations, and were utilized to advantage in the progress of civilization.” (Grinstein, 1952; p. 334)

Similarly, Dawkins (1982) emphasizes the evolutionary constraints on perfection of the organisms in terms of economical use of bodily resources:

“For a bird, resources spent on making breast muscles for powering wings are resources that could have been spent on making eggs. An enlarged brain would permit a finer tuning of behavior to environmental details, past and present, but at a cost of an enlarged head, which means extra weight at the front end of the body, which in turn necessitates a larger tail for aerodynamic stability, which in turn…” (p. 47)

The acquisition of fire enabled prehistoric man to maximize what can be achieved by limited bodily/evolutionary resources. Therefore, the excessive evolutionary yield was made available, to be invested in the evolution of the rest of the body and the brain. Clark and Harris (1985) suggest that in early hominid life fire was particularly used as a lighting source and as a means of protection from dangerous animals, providing support for these evolutionary processes. Evolutionary expenditures such as achieving muscular fitness to confront predators or growing thick fur to stay warm would have gradually lost their priority. Exponentially, fire would have significantly improved the nutritional properties of both plant and animal foods (Stahl et al., 1984), increasing the overall energy efficiency of the hominid as a complete biological entity. The long term impacts of the smallest advantages created by acquiring an advantageous behavioral strategy have been suggested by Slavin and Kriegman (1992). These authors showed that a behavioral strategy which creates 1/10,000 advantage in reproductive fitness would lead to 692,981 more offspring in just 99 generations (p. 288). In a similar fashion, the evolutionary resource savings achieved by the acquisition of fire were then invested in to the evolution of a more complex cerebral cortex. This diverted the course of evolution, separating *Homo sapiens* from other animals in terms of complexity of psychological processes which emerged.

Sublimation provides two-fold reinforcements for human evolution. The detrimental effects of close interbreeding were probably resolved. Sublimation created opportunities to maximize individual gains while at the same time equipping prehistoric man against surrounding dangers. Sublimation as a deviant strategy (novel strategies which improve cost-efficiency of tool use even further) increased the likelihood of sexual reproduction for the members within a social horde other than the alpha male, by introducing an alternative to avoidance strategies which guide male members into competition to reach to an optimum size and fitness. Individuals using any deviant strategy (any behavioral modification that leads to higher cost efficiency relative to what has been achieved by the rest of the population) maximized their gains and these strategies which would have higher reproduction fitness would spread out in the population until they reach to an equilibrium point, becoming an ESS. From this time point, every little invention created evolutionary resourceful savings that are indirectly invested in the evolution of the cortex. The inventors maximized individual gains through savings of evolutionary resources. Each individual became a deviant micro-strategy spreading their skills until a new equilibrium point was established across the population, exponentially contributing to the advancement of civilization.

## Limitations

The current discussion inevitably suffers from the very same limitation Freud faced nearly 100 years ago. What one may call the “socio-moral anthropology” cannot dissociate itself from the generation of hypotheses which cannot be empirically tested due to a lack of written evidence dating back to the Pleistocene or any other Prehistoric period. Therefore, this account may be perceived as speculative by the readers. Even though it is possible to foresee that another 100 years may not be enough to increase the accuracy of reconstructions such as the one proposed here, advances in related fields such as evolutionary biology, evolutionary economics, anthropology, cognitive neuroscience, and primate research may help proposing more up-to-date models. It is highly likely that the new findings emerging in these fields will shed light to our origins as a moral agent.

## Conclusion

It is commonly acknowledged that Freud wanted to ground psychoanalytical theory within the earth sciences. However, this article suggests that theoretical discussions can be advanced by considering contributions from the life sciences. Freud's views on the primal horde are taken into consideration in the light of modern evolutionary biology and the acquisition of fire in the Prehistoric times. The latter is regarded as the most significant achievement which might have led to critical evolutionary resourceful savings. The possible role of sublimation in the acquisition of fire has been explored and a link between sublimation and the ultimate evolution of cerebral cortex is proposed. The possible role of this process in explaining the gradual formation of the superego has been discussed. The present approach somewhat contradicts Freud's hypothesis about the origins of conscience and superego in the primal horde which is presented as if these have emerged instantly. The discussion presented here implicitly suggests that Freud's primal horde was already far ahead of similar animal hordes on the evolutionary timeline. The presence of self-conscious moral emotions (Tangney et al., 2007) such as guilt suggests that the members of the primal horde achieved a transition from purely biological mechanisms to other psychological mechanisms/motivators. The author proposes that further theoretical discussions could accredit psychoanalytical theory within both life and earth sciences and contribute to the production of testable empirical hypotheses in the future.

### Conflict of interest statement

The author declares that the research was conducted in the absence of any commercial or financial relationships that could be construed as a potential conflict of interest.

## Footnotes

^1^An evolutionary stable strategy is defined as a strategy which, if most members of a population adopt it, can not be improved by an alternative strategy (Dawkins, 1976). It can be claimed that an ESS is immune to any kind of deviant strategy trying to maximize individual gains in any population.

^2^Dawkins (1976) introduces the memes as complementary replicators which carry ideas, customs and other relevant cultural legacies. Just as genes, they are subjected to natural selection and their life course depends on their effectiveness in adaptation to the culture and the time.

## References

[B1] American Psychiatric Association (2000). Diagnostic and Statistical Manual of Mental Disorders, (4th Edn, text rev.). Washington, DC

[B1a] Alexander R. D. (1987). The Biology of Moral Systems. Chicago: Transaction Books

[B2] Bachelard G. (1964). The Psychoanalysis of Fire, Trans. Ed Ross C. M. Boston, MA: Beacon press

[B3] Banich M. T. Milham M. P. Atchley R. Cohen N. J. Webb A. Wszalek T. (2000). fMRI studies of Stroop tasks reveal unique roles of anterior and posterior brain systems in attentional selection. J. Cogn. Neurosci. 12, 988–1000 10.1162/08989290051137521 11177419

[B4] Bazzan A. L. C. Bordini R. H. Campbell J. A. (2002). Evolution of agents with moral sentiments in an iterated prisoner's Dilemma exercise in Game Theory and Decision Theory in Agent-based Systems, eds Parsons S. Gmytrasiewicz P. Wooldridge M. (Springer), 43–64 10.1007/978-1-4615-1107-6_3

[B5] Bellomo R. V. (1994). Methods of determining early hominid behavioural activities associated with the controlled use of fire at FxJj 20 Main, Koobi Fora, Kenya. J. Hum. Evol. 27, 173–195 10.1006/jhev.1994.1041

[B6] Boag S. (2007). ‘Real processes’ and the explanatory status of repression and inhibition. Philos. Psychol. 20, 375–392 10.1080/09515080701361173

[B7] Boly M. Seth A. K. Wilke M. Ingmundson P. Baars B. Laureys S. (2013). Consciousness in humans and non-human animals: recent advances and future directions. Front. Psychol. 4:625 10.3389/fpsyg.2013.00625 24198791 PMC3814086

[B8] Bowlby J. (1977). The making and breaking of affectional bonds. I. Aetiology and psychopathology in the light of attachment theory. An expanded version of the Fiftieth Maudsley Lecture, delivered before the Royal College of Psychiatrists, 19 November 1976. Br. J. Psychiatry 130, 201–210 10.1192/bjp.130.3.201 843768

[B9] Bretherton I. (1985). Attachment theory: retrospect and prospect. Monogr. Soc. Res. Child Dev. 3–35 10.2307/3333824

[B10] Brinker J. K. Dozois D. J. A. (2009). Ruminative thought style and depressed mood. J. Clin. Psychol. 65, 1–19 10.1002/jclp.20542 19048597

[B11] Brodmann K. (1909). Vergleichende Lokalisationslehre der Grosshirnrinde: in ihren Prinzipien dargestellt auf Grund des Zellenbaues. Leipzig: Ja Barth

[B12] Burton F. D. (2011). Fire: The Spark that Ignited Human Evolution. New Mexico: UNM Press

[B13] Clark J. D. Harris J. W. K. (1985). Fire and its roles in early hominid lifeways. Afr. Archeol. Rev. 3, 3–27 10.1007/BF01117453

[B14] Daly M. Wilson M. (1988). Evolutionary social psychology and family homicide. Science 242, 519–524 10.1126/science.3175672 3175672

[B15] Dawkins R. (1976). The Selfish Gene. Oxford: Oxford University Press

[B16] Dawkins R. (1982). The Extended Phenotype. Oxford: Oxford University Press

[B17] Deri F. (1939). On sublimation. Psychoanal. Q. 8, 325–334

[B18] Erlenmeyer E. H. (1932). Note on Freud's hypothesis regarding the taming of fire. Int. J. Psychoanal. 13, 411–413

[B19] Freud S. (1900). Interpretation of dreams. SE 4–5

[B20] Freud S. (1913). Totem and taboo. SE 13, 1–161

[B21] Freud S. (1915a). On Narcissism. SE 14, 94–110

[B22] Freud S. (1915b). Instincts and their vicissitudes. SE 14, 111–140

[B23] Freud S. (1917). Mourning and melancholia. SE 14, 243–258

[B24] Freud S. (1920). Beyond pleasure principle. SE 18, 7–64

[B25] Freud S. (1921). The group psychology and the analysis of the ego. SE 18, 65–143 625511

[B26] Freud S. (1923). The Ego and the Id. SE 19, 3–66 14198857

[B27] Freud S. (1924). The dissolution of the oedipus complex. SE 19, 171–180

[B28] Freud S. (1926). Inhibitions, symptoms and anxiety. SE 20, 77–174

[B29] Freud S. (1930a). Civilization and its discontents. SE 21, 64–145

[B30] Freud S. (1930b). Das Unbehagen in der Kultur. Wieni: Internationaler Psychoanalytischer Verlag, 1–141

[B31] Freud S. (1932). The acquisition and control of fire. SE 22, 187–193

[B32] Gemes K. (2009). Freud and Nietzsche on sublimation. J. Nietzsche Stud. 38, 38–59 10.1353/nie.0.00481108066

[B33] Gershenowitz H. (1978). The influence of Lamarckism on the development of Freud's psychoanalytic theory. Dev. Freud's Psychoanal. Theory 14, 105–114 11611066

[B34] Giedd J. N. (2004). Adolescent brain development: vulnerabilities and opportunities. Ann. N.Y. Acad. Sci. 1021, 77–85 10.1196/annals.1308.009 15251877

[B35] Goren-Inbar N. Alperson N. Kislev M. E. Simchoni O. Melamed Y. Ben-Nun A. (2004). Evidence of hominin control of fire at Gesher Benot Ya'aqov, Israel. Science 304, 725–727 10.1126/science.1095443 15118160

[B36] Gould J. S. (1977). Ontogeny and Phylogeny. Boston, MA: Harvard University Press

[B37] Greene J. D. Sommerville R. B. Nystrom L. E. Darley J. M. Cohen J. D. (2001). An fMRI investigation of emotional engagement in moral judgement. Science 293, 2105–2108 10.1126/science.1062872 11557895

[B38] Grinstein A. (1952). Stages in the development of control over fire. Int. J. Psychoanal. 33, 416–420 12999362

[B39] Holden C. (1979). Paul MacLean and the triune brain. Science 204, 1066–1068 10.1126/science.377485 377485

[B40] Holmes J. (2011). Superego: an attachment perspective. Int. J. Psychoanal. 92, 1221–1240 10.1111/j.1745-8315.2011.00411.x 22014367

[B41] Klein M. (1997). The Psychoanalysis of Children. London: Random House

[B42] Knoch D. Nitsche M. A. Fischbacher U. Eisenegger C. Pascual-Leone A. Fehr E. (2008). Studying the neurobiology of social interaction with transcranial direct current stimulation—the example of punishing unfairness. Cereb. Cortex 18, 1987–1990 10.1093/cercor/bhm237 18158325

[B43] Lantos B. (1958). The two genetic derivations of aggression with reference to sublimation and neutralization. Int. J. Psychoanal. 39, 116–120 13574947

[B44] MacLean P. (1985). Evolutionary psychiatry and the triune brain. Psychol. Med. 15, 219–221 10.1017/S0033291700023485 4023126

[B45] Mega S. M. Cummings J. L. Solloway S. Malloy P. (1997). The limbic system: an anatomic, phylogenetic, and clinical perspective. J. Neuropsychiatry Clin. Neurosci. 9, 315–330 9276837 10.1176/jnp.9.3.315

[B46] Mills J. (2004). Clarifications on trieb: Freud's theory of motivation reinstated. Psychoanal. Psychol. 21, 673–677 10.1037/0736-9735.21.4.673

[B47] Moll J. Zahn R. de Oliveira-Souza R. Kruger F. Grafman J. (2005). Opinion: the neural basis of human moral cognition. Nat. Rev. Neurosci. 6, 799–810 10.1038/nrn1768 16276356

[B48] Nesse R. M. (1990). The evolutionary functions of repression and the ego defences. J. Am. Acad. Psychoanal. 18, 260–285 2387763 10.1521/jaap.1.1990.18.2.260

[B49] Nesse R. M. Lloyd A. T. (1992). Evolution of psychodynamic mechanisms in The Adapted Mind, eds Barkow J. H. Cosmides L. Tooby J. (New York, NY: Oxford University Press), 601–624

[B50] Nietzsche F. W. (1878). Human, All Too Human: A Book for Free Spirits. Trans eds Faber M. Lehmann S. Lincoln: University of Nebraska Press, 1984, 43–44, 67–68.

[B51] O'Connor L. E. Berry J. W. Lewis T. Mulherin K. Crisostomo P. S. (2007). Empathy and depression: the moral system on overdrive in Empathy and Mental Illness, eds Farrow T. Woodruff P. W. R. (New York, NY: Cambridge University Press), 49–75

[B52] Payne S. M. (1927). Observations on the formation and function of the super-ego in normal and abnormal psychological states1. Br. J. Med. Psychol. 7, 73–87 10.1111/j.2044-8341.1927.tb00647.x

[B53] Pulcu E. Zahn R. Elliott R. (2013). The role of self-blaming moral emotions in major depression and their impact on social-economical decision making. Front. Psychol. 4:310 10.3389/fpsyg.2013.00310 23750148 PMC3670430

[B55] Schultz W. (2004). Neural coding of basic reward terms of animal learning theory, game theory, microeconomics and behavioural ecology. Curr. Opin. Neurobiol. 14, 139–147 10.1016/j.conb.2004.03.017 15082317

[B56] Scott A. C. Glasspool I. J. (2006). The diversification of Paleozoic fire systems and fluctuations in atmospheric oxygen concentration. Proc. Natl. Acad. Sci. U.S.A. 103, 10861 10.1073/pnas.0604090103 16832054 PMC1544139

[B57] Seely W. W. Merkle F. T. Gaus S. E. Craig A. D. Allman J. M. Hof P. R. (2012). Distinctive neurons of the anterior cingulate and frontoinsular cortex: a historical perspective. Cereb. Cortex 22, 245–250 10.1093/cercor/bhr005 21653703

[B57a] Ségalen L. Lee-Thorp J. A. Cerling T. (2007). Timing of C4 grass expansion across sub-Saharan Africa. J. Hum. Evol. 53, 549–559 10.1016/j.jhevol.2006.12.010 17905413

[B58] Sekine K. Rose M. L. (2013). The relationship of aphasia type and gesture production in people with aphasia. Am. J. Speech Lang. Pathol. 22, 662 10.1044/1058-0360(2013/12-0030) 24018695

[B59] Slavin M. O. Kriegman D. (1992). The Adaptive Design of the Human Psyche: Psychoanalysis, Evolutionary Biology, and the Therapeutic Process. New York, NY: Guilford Press

[B59a] Stahl A. B. Dunbar R. I. M. Homewood K. Ikawa-Smith F. Kortlandt A. McGrew W. C. (1984). Hominid dietary selection before fire [and Comments and Reply]. Curr. Anthropol. 25, 151–168

[B60] Stone L. (1979). Remarks on certain unique conditions of human aggression (the hand, speech, and the use of fire). J. Am. Psychoanal. Assoc. 27, 27–63 489884 10.1177/000306517902700102

[B61] Tangney J. P. (2002). Self-conscious moral emotions: the self as a moral guide in Self and Motivation: Emerging Psychological Perspectives, eds Tesser A. Stapel D. A. Wood J. V. (Washington, DC: American Psychological Association), 97–117

[B62] Tangney J. P. Stuewig J. Mashek D. (2007). Moral emotions and moral behavior. Annu. Rev. Psychol. 58, 345 10.1146/annurev.psych.56.091103.070145 16953797 PMC3083636

[B62a] Trivers R. L. (1985). Social Evolution. Menlo Park, CA: Benjamin/Cummings

[B63] van Schaik C. P. Deaner R. O. Merrill M. Y. (1999). The conditions for tool use in primates: implications for the evolution of material culture. J. Hum. Evol. 36, 719–741 10.1006/jhev.1999.0304 10330335

[B64] Whelan R. J. (1995). The Ecology of Fire. Cambridge: Cambridge University Press

[B65] Whitcome K. K. Shapiro L. J. Lieberman D. E. (2007). Fetal load and the evolution of lumbar lordosis in bipedal hominins. Nature 450, 1075–1078 10.1038/nature06342 18075592

[B66] Wrangham R. W. Jones J. H. Laden G. Pilbeam D. Conklin-Brittain N. (1999). The raw and the stolen: cooking and the ecology of human origins. Curr. Anthropol. 40, 567–594 10.1086/300083 10539941

[B67] Young L. J. Wang Z. X. (2004). The neurobiology of pair bonding. Nat. Neurosci. 7, 1048–1054 10.1038/nn1327 15452576

